# Supplementary material for: Distinct subnetworks of the mouse anterior thalamic nuclei
Source: Nat Commun. 2025 Jul 1;16:6018. doi: 10.1038/s41467-025-60774-6 (PMC12216302; doi:10.1038/s41467-025-60774-6)
Supplement: Supplementary file 1 — Supplementary Information file [file 41467_2025_60774_MOESM1_ESM.pdf]

Supplementary Information

Distinct subnetworks of the mouse anterior thalamic nuclei

Houri Hintiryan[1*], Mitchell Rudd[1#], Sumit Nanda[1#], Adriana E. Gutierrez[1], Darrick Lo[1], Tyler Boesen[1], Luis Garcia[1], Jiandong Sun[1], Christian Estrada[1], Hyun-Seung Mun[1], Seita Yamashita[1], Yeji E. Han[1], Ian Bowman[1], Lin Gou[1], Chunru Cao[1], Jennifer Gonzalez[1], Keivan Moradi[1], Qiuying Zhao[1], Inga Yenokian[1,5], Aishwarya Dev[1], Brian Zingg[1], Hanpeng Xu[1], Qing Xue[1], Muye Zhu[1,6], Lijuan Liu[2,3], Xin Chen[2], Zhixi Yun[2], Hanchuan Peng[4], Nicholas N. Foster[1], Hong-Wei Dong[1*]

Affiliations

1. UCLA Brain Research & Artificial Intelligence Nexus (B.R.A.I.N.), Department of Neurobiology, David Geffen School of Medicine at UCLA, University of California Los Angeles, Los Angeles, CA 90089, USA

2. New Cornerstone Science Laboratory, SEU-ALLEN Joint Center, Institute for Brain and Intelligence, Southeast University, Nanjing, China

3. School of Biological Science & Medical Engineering, Southeast University, Nanjing, China

4. Shanghai Academy of Natural Sciences (SANS), Fudan University, Shanghai, China

# These authors made equal contributions
5 Current address: Cedars-Sinai Medical Center, Los Angeles, CA, USA
6 Current address: Google, Boulder, CO, USA
* Corresponding authors: HHintiryan@mednet.ucla.edu and HongWeiD@mednet.ucla.edu

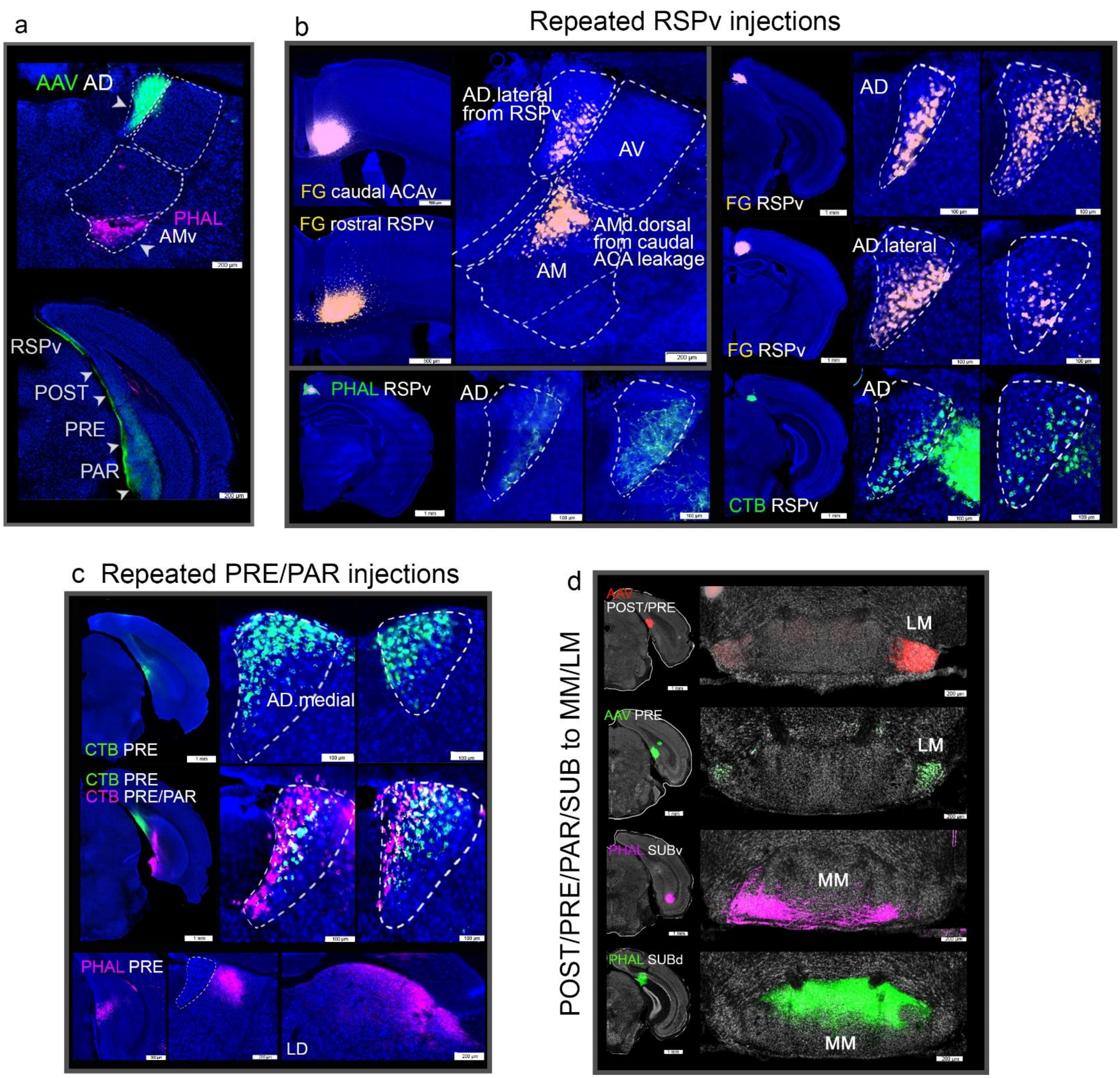

Supplementary Fig. 1, related to Fig. 2. AD connections

a. An anterograde AAV tracer injection in the AD labels all layers of the POST/PRE/PAR (AD→POST/PRE/PAR) and superficial layers of the RSPv (AD→RSPv). **b.** Repeated injections made across different levels of the RSPv consistently label primarily the AD.lateral. One FG injection that covers both the caudal ACAv and the rostral RSPv labels both the AD.lateral and the AMd.dorsal domains validating the AD.medial→RSPv and the AMd.dorsal→caudal ACAv connections. **c.** Repeated tracer injections in PRE and PAR consistently label AD.medial. Labeling in the LD is shown as verification of the PHAL PRE injection (see Supplementary Table 2 for number of repeated injections made in each ROI). **d.** Anterograde tracer injections in POST, PRE, PAR, and SUB show their distinct projections to the MM and LM, which were used to verify locations of the hippocampal injection sites. See Supplementary Table 2 for structure abbreviations.

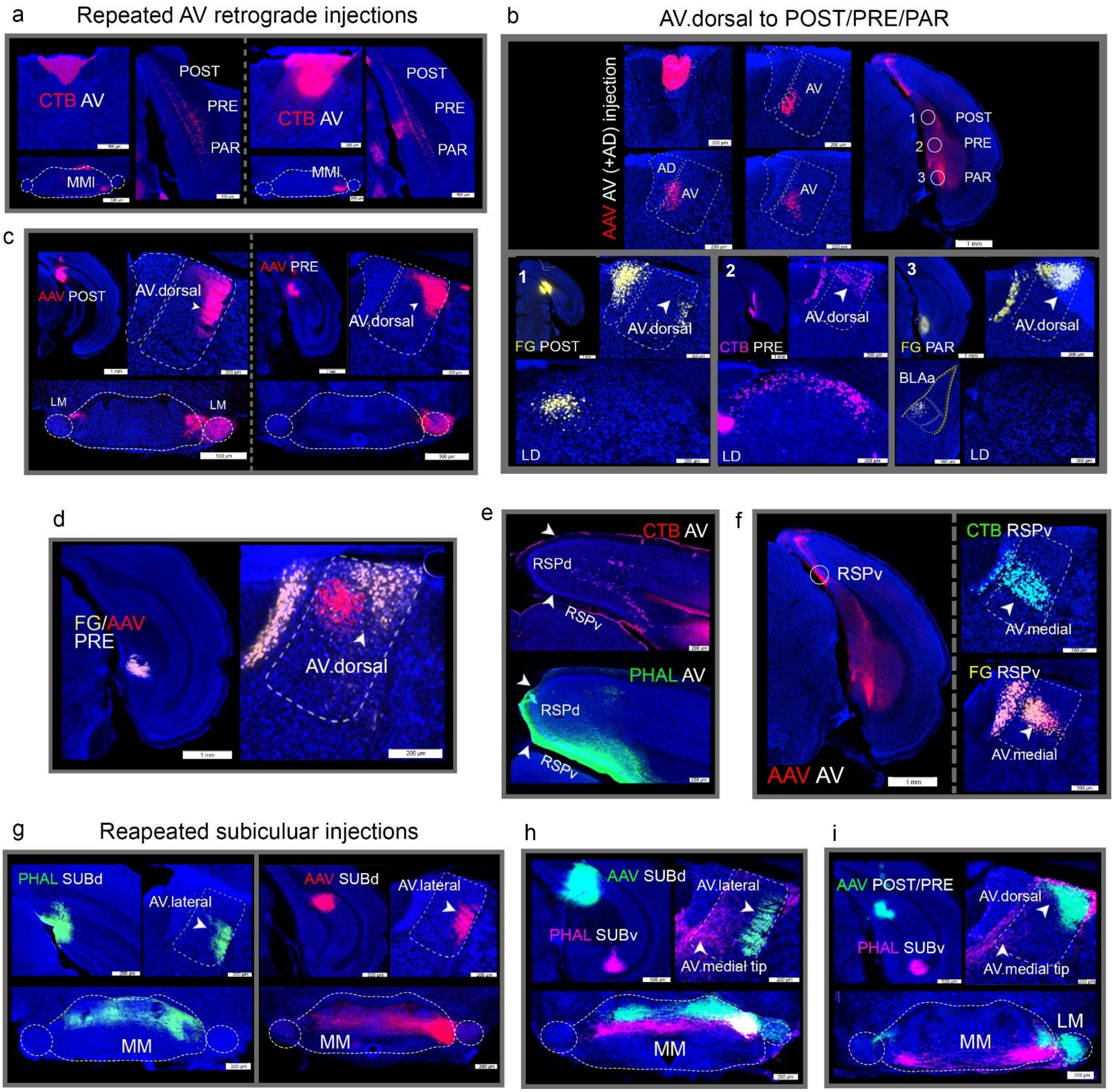
a
Repeated AV retrograde injections
CTB AV
POST
PRE
PAR
MMl
b
AV.dorsal to POST/PRE/PAR
AAV AV (+AD) injection
AV
AD
1
2
3
FG POST
AV.dorsal
LD
CTB PRE
FG PAR
BLAa
c
AAV POST
AAV PRE
LM
d
FG/AAV PRE
e
CTB AV
PHAL AV
RSPd
RSPv
f
AAV AV
CTB RSPv
FG RSPv
AV.medial
g
Reapeated subicular injections
PHAL SUBd
AAV SUBd
AV.lateral
MM
h
AAV SUBd
PHAL SUBv
AV.medial tip
i
AAV POST/PRE

Supplementary Fig. 2, related to Fig. 3. AV connections

a. Repeated AV retrograde tracer injections consistently label neurons in the deep layers of POST, PRE, and PAR (see Supplementary Table 2 for number of repeated injections made in each ROI). Labeled neurons in the MMl shown as verification of AV injection location. **b.** Retrograde tracer injections were placed in regions of the axon terminals resulting from an AV (plus AD) anterograde tracer injection. CTB injections in the POST (1), PRE (2), and PAR (3) labels the AV.dorsal (and AD.medial) validating the AV and AD domain specific connections with each of the ROIs. In the bottom panels, retrogradely labeled cells in the LD (for POST, PRE, PAR) and BLA (for PAR) are shown to verify the tracer injection locations. For example, the PAR receives input from the BLA and none from the LD, while the POST and PRE receive input from different groups of neurons in the LD and none from the BLA. **c.** Anterograde AAV injections in the POST and PRE show projections specifically to AV.dorsal. Projections to MM and LM shown injection site location verification. **d.** An AAV/FG co-injection in PRE confirms the unidirectional AD.medial→PRE and the bidirectional AV.dorsal↔PRE connections. LD labeling is shown to verify PRE injection locations. **e.** CTB (top) and PHAL (bottom) AV injections show bidirectional connections with RSPd and RSPv (AV↔RSPd/v). **f.** AV AAV injection shows projections to RSPv. Dashed circle denotes location of the CTB and FG injections that label neurons in the AV.medial validating the AV.medial→RSPv connection. **g-i.** Several anterograde tracer injections in the SUBd and SUBv consistently show SUBv→AV.medial and SUBd→AV.lateral connections. See Supplementary Table 2 for structure abbreviations.

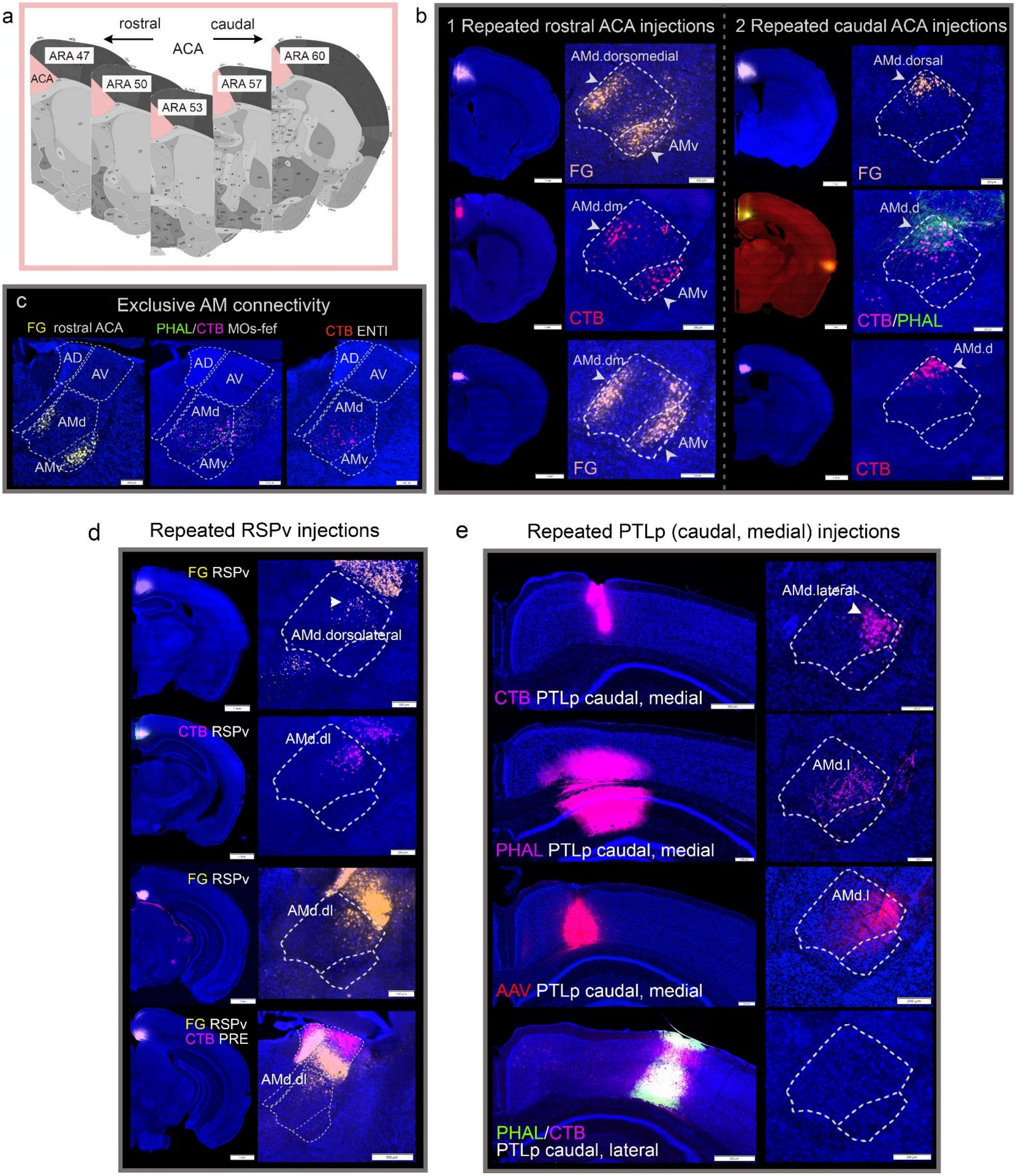
a
rostral
caudal
ACA
ARA 47
ACA
ARA 50
ARA 53
ARA 57
ARA 60
b
1 Repeated rostral ACA injections
2 Repeated caudal ACA injections
AMd.dorsomedial
AMv
FG
AMd.dorsal
FG
AMd.dm
AMv
CTB
AMd.d
CTB/PHAL
AMd.dm
AMv
FG
AMd.d
CTB
c
Exclusive AM connectivity
FG rostral ACA
PHAL/CTB MOs-fef
CTB ENTl
AD
AV
AMd
AMv
d
Repeated RSPv injections
FG RSPv
AMd.dorsolateral
CTB RSPv
AMd.dl
FG RSPv
AMd.dl
FG RSPv
CTB PRE
AMd.dl
e
Repeated PTLp (caudal, medial) injections
CTB PTLp caudal, medial
AMd.lateral
PHAL PTLp caudal, medial
AMd.l
AAV PTLp caudal, medial
AMd.l
PHAL/CTB
PTLp caudal, lateral

Supplementary Fig. 3, related to Figs. 4 and 5. AMd.dorsal, AMd.dorsolateral, and AMd.lateral domain connections

a. ARA atlas levels showing the approximate boundary between rostral (<ARA 53) and caudal (>ARA 53) ACA based on their connections with the AM. **b.** Numerous injections made in the rostral ACA (left) and caudal ACA (right) substantiate the AMd.dorsomedial↔rostral ACA and AMd.dorsal↔caudal ACA connectivity. **c.** The ACA, MOs-fef, and ENTl connectivity with the ATN are exclusively through the AM. Tracer injections in each of these ROIs label terminals or cells only in the AM and not in the AD or AV. **d.** Several retrograde injections in the RSPv consistently label neurons in the AMd.dorsolateral. In the final panel, a FG RSPv and a CTB PRE injection were made in the same brain. This case shows the AMd.dorsolateral→RSPv connection, but also the AV.medial→RSPv, AD.lateral→RSPv, AV.dorsal→PRE, and AD.medial→PRE connections. Note the absence of CTB labels in the AM. **e.** Many injections in the PTLp (caudal, medial part) label the AMd.lateral, while in the final panel, a PHAL/CTB co-injection in the PTLp (caudal, lateral part) does not show any labeled axon terminals or cells in the AM validating the specific AMd.lateral↔PTLp (caudal, medial) connection. See Supplementary Table 2 for structure abbreviations.

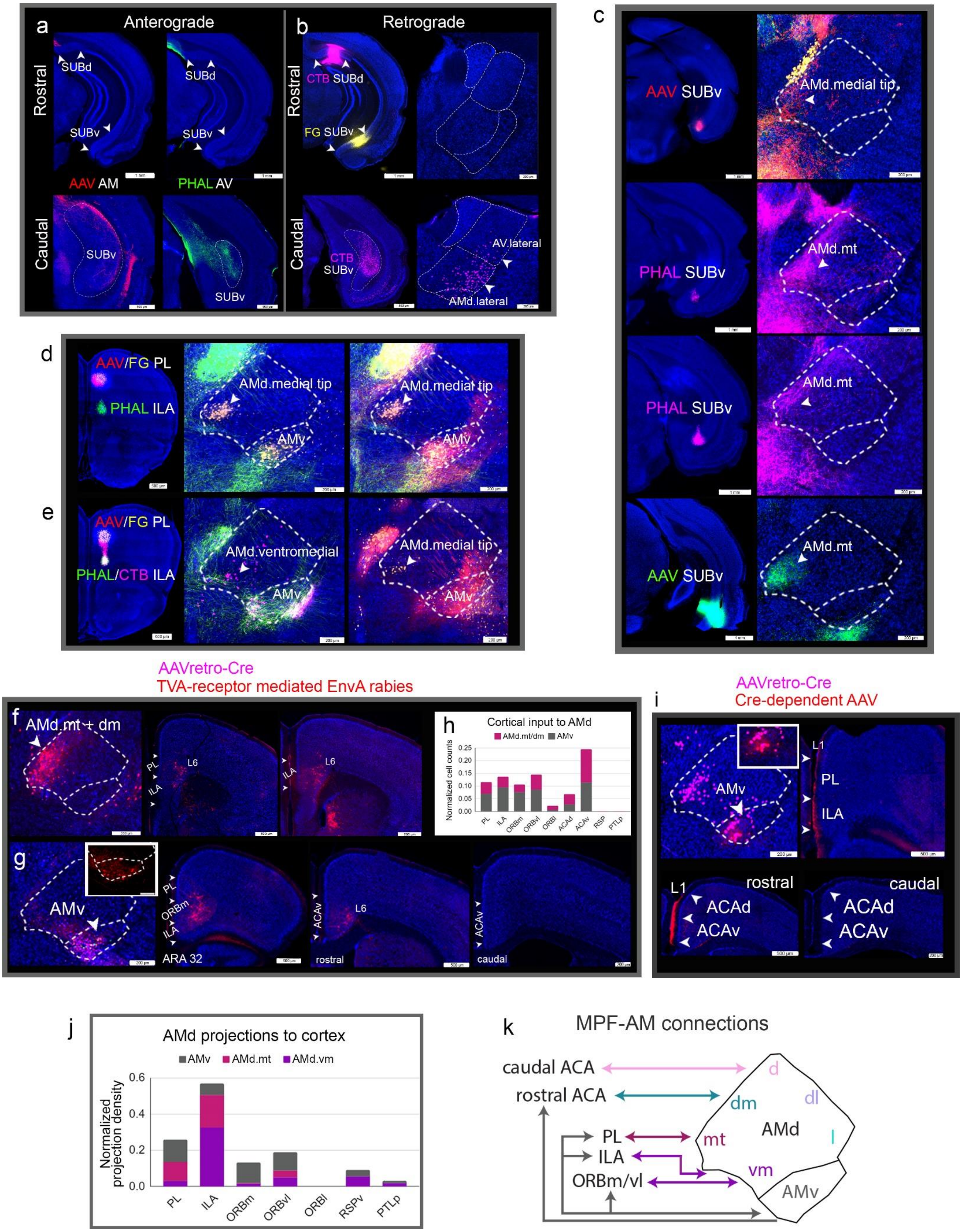
a
Anterograde
Rostral
SUBd
SUBv
AAV AM
PHAL AV
Caudal
SUBv
b
Retrograde
CTB SUBd
FG SUBv
CTB SUBv
AV.lateral
AMd.lateral
c
AAV SUBv
AMd.medial tip
PHAL SUBv
AMd.mt
AAV SUBv
d
AAV/FG PL
PHAL ILA
AMd.medial tip
AMv
e
AAV/FG PL
PHAL/CTB ILA
AMd.ventromedial
AMd.medial tip
AAVretro-Cre
TVA-receptor mediated EnvA rabies
f
AMd.mt + dm
PL
ILA
L6
h
Cortical input to AMd
AMd.mt/dm
AMv
Normalized cell counts
0.25
0.20
0.15
0.10
0.05
0.00
PL
ILA
ORBm
ORBvl
ORBl
ACAd
ACAv
RSP
PTLp
g
AMv
ORBm
ARA 32
ACAv
rostral
caudal
i
AAVretro-Cre
Cre-dependent AAV
L1
PL
ILA
ACAd
ACAv
j
AMd projections to cortex
AMv
AMd.mt
AMd.vm
Normalized projection density
0.6
0.4
0.2
0.0
RSPv
PTLp
k
MPF-AM connections
caudal ACA
rostral ACA
PL
ILA
ORBm/vl
d
dm
dl
mt
AMd
l
vm
AMv

Supplementary Fig. 4, related to Fig. 6. AMd.medial tip, AMd.ventromedial, and AMv connections
a. Projections from the AM and AV to SUB are sparse and are to a very specific region within the caudal SUB. Anterograde tracer injections in the AM and AV do not label the SUB in rostral sections, but instead label caudal parts of the SUBv albeit with different patterns. **b**. Retrograde tracer injections placed in the SUBd and SUBv in rostral levels show no labeled neurons in the AV or AM, while a CTB injection in the caudal SUBv labels neurons primarily in the AV.lateral and AMd.lateral domains. **c.** Repeated SUBv injections validate the SUBv→AMd.medial projections (see Supplementary Table 2 for number of repeated injections made in each ROI). **d-e.** Distinction between AMd.medial tip and AMd.ventromedial domains. **d.** AAV/FG co-injection in PL shows AMd.medial tip↔PL (but also AMv↔PL). **e.** Anterograde/retrograde co-injections in the PL and ILA in the same brain show AMd.medial tip↔PL, AMd.ventromedial↔ILA, and AMv↔ILA/PL connections. **f.** A Cre-dependent TVA receptor mediated rabies injection in the AMv (AAVretro-Cre injection in the MPF) back labels neurons in the PL, ORBm, ILA, and rostral ACAv (layer VI), substantiating inputs to AMv from these regions. Note the absence of labels in the caudal ACAv. **g.** A Cre-dependent TVA receptor mediated rabies injection in the AMd.medial tip back labels cells in the PL and ILA confirming input from these regions to AMd.medial tip/dorsomedial (AAVretro-Cre injection in the MPF). **h.** Quantification of the data from f-g shows the distinct connections of AMd.medial tip versus the AMv. **i.** Cre-dependent tracing of AMv neurons (AAVretro-Cre in MPF) shows projections to layer 1 of PL, ILA, and rostral ACA. Observe the lack of labeling in the caudal ACA. **j.** Quantification of projections from the AMv, AMd.medial tip, and AMd.ventromedial domains showing their relative projections. Note the stronger projections from AMv and AMd.medial tip to PL, AMd.ventromedial to ILA, AMv to ORBm/vl (but not ORBl), and overall fewer projections to RSP and PTLp from these regions. This bar graph validates all the data presented for each of these domains. **k.** Summary of connections among the MPF and the AMd domains and AMv.

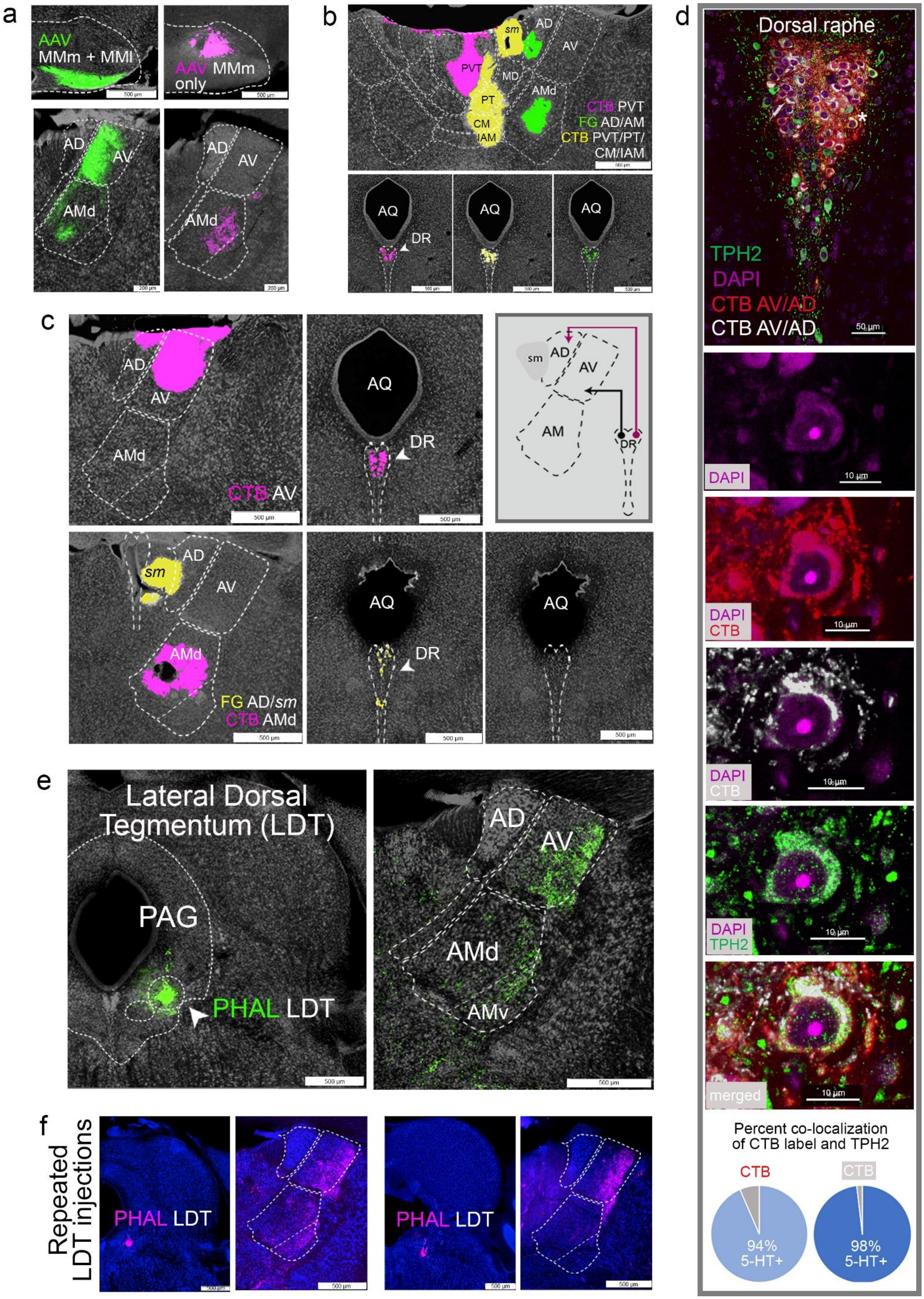
a
AAV
MMm + MMl
AAV MMm
only
500 µm
AD
AV
AMd
200 µm
b
sm
AD
AV
PVT
MD
PT
AMd
CM
IAM
CTB PVT
FG AD/AM
CTB PVT/PT/
CM/IAM
AQ
DR
500 µm
c
AD
AV
AMd
CTB AV
AQ
DR
sm
AM
sm
FG AD/sm
CTB AMd
d
Dorsal raphe
TPH2
DAPI
CTB AV/AD
CTB AV/AD
50 µm
DAPI
10 µm
DAPI
CTB
DAPI
TPH2
merged
Percent co-localization
of CTB label and TPH2
CTB
CTB
94%
5-HT+
98%
5-HT+
e
Lateral Dorsal
Tegmentum (LDT)
PAG
PHAL LDT
AD
AV
AMd
AMv
f
Repeated
LDT injections
PHAL LDT
PHAL LDT

Supplementary Fig. 5, related to Fig. 9. Connections of the ATN with the dorsal raphe (DR) and lateral dorsal tegmental nucleus

a. An AAV injection that spans both the MMm and MMl labels both the AM and AV, while one restricted to the MMm labels only the AM. **b.** Retrograde tracer injections in PVT (CTB, pink), PT, CM, IAM (CTB, yellow) show the well documented DR projections to the midline and intralaminar thalamic nuclei. A retrograde tracer in the AM, with leakage into the AD, also shows retrogradely labeled cells in the DR, suggesting a DR→AM/AD connection, but a circumscribed AM retrograde injection does not back label DR cells in **c**, which also shows DR→AV and confirms the DR→AD projection. Together, the data suggest DR→AD/AV connections. **d.** Most AV/AD projecting neurons in the DR are serotonergic. In a single animal, two separate CTB tracer injections were made in the AV/AD (CTB 561, red; CTB 647, white). Both injections labeled neurons in the DR. 50 µm sections through the DR were stained for tryptophan hydroxylase (TPH2) to identify serotonin positive cells. A 60x confocal image (0.5 µm z) shows an overview of the DR with CTB and TPH2 labeled cells. A magnified image of a cell co-labeled with CTB and TPH2 is shown. Manual counts of CTB labeled cells and TPH2+ cells are revealed that 94% and 98% of the CTB labeled DR cells were also positive for TPH2, which are visualized in pie charts on the bottom. **e.** A PHAL injection in the LDT labels axons in the AV and AM. **f.** Given the small size of the LDT, repeated anterograde injections were made, which consistently labeled axons in the AV and AM (see Supplementary Table 2 for number of repeated injections made in each ROI). The LDT does not project to MM, which is also shown. See Supplementary Table 2 for structure abbreviations.

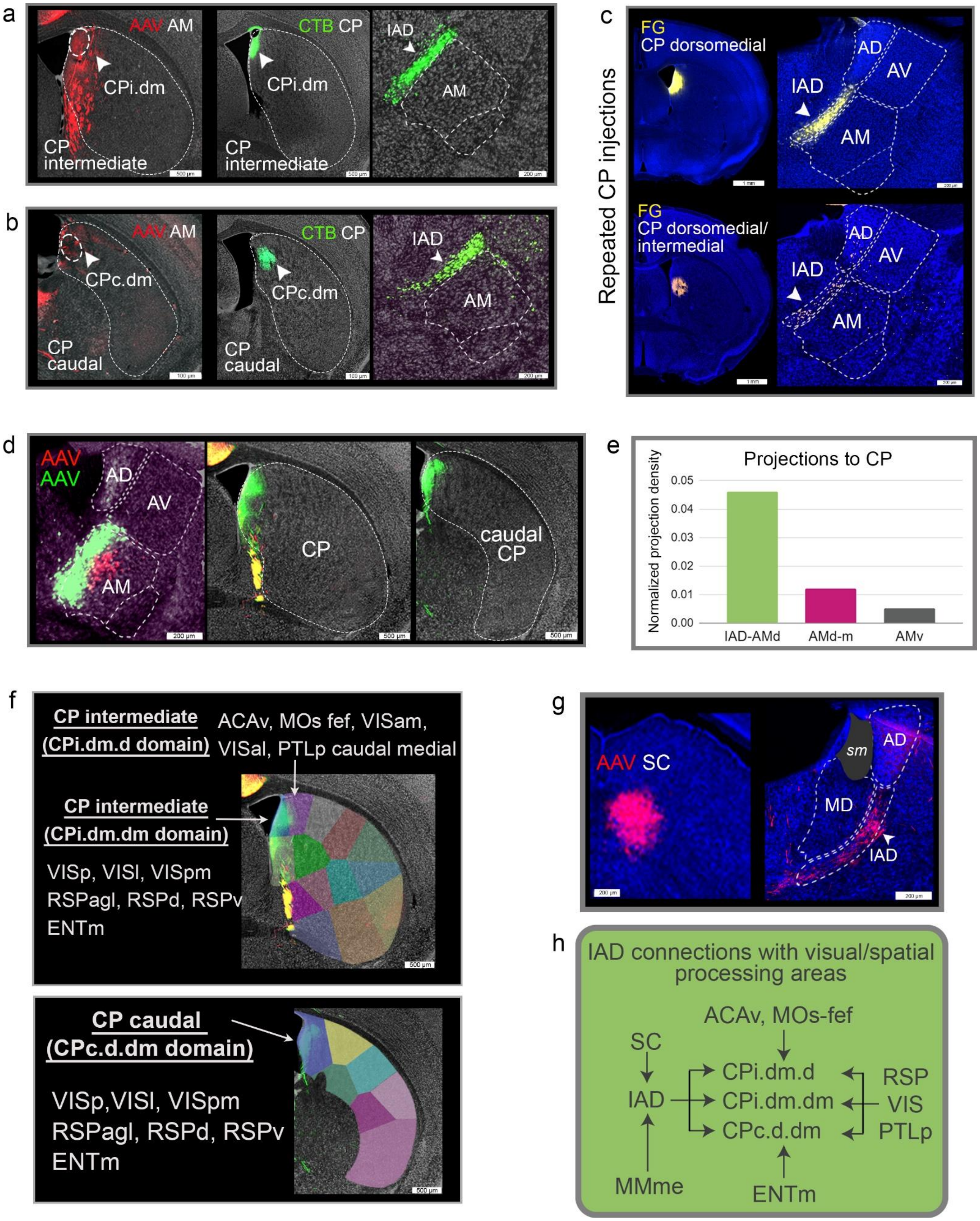
a
AAV AM
CPi.dm
CP intermediate
CTB CP
CPi.dm
CP intermediate
IAD
AM
b
AAV AM
CPc.dm
CP caudal
CTB CP
CPc.dm
CP caudal
IAD
AM
c
Repeated CP injections
FG
CP dorsomedial
AD
AV
IAD
AM
FG
CP dorsomedial/ intermediate
AD
AV
IAD
AM
d
AAV
AAV
AD
AV
AM
CP
caudal CP
e
Projections to CP
Normalized projection density
0.05
0.04
0.03
0.02
0.01
0.00
IAD-AMd
AMd-m
AMv
f
CP intermediate (CPi.dm.d domain)
ACAv, MOs fef, VISam, VISal, PTLp caudal medial
CP intermediate (CPi.dm.dm domain)
VISp, VISl, VISpm
RSPagl, RSPd, RSPv
ENTm
CP caudal (CPc.d.dm domain)
VISp,VISl, VISpm
RSPagl, RSPd, RSPv
ENTm
g
AAV SC
sm
AD
MD
IAD
h
IAD connections with visual/spatial processing areas
ACAv, MOs-fef
SC
IAD
CPi.dm.d
CPi.dm.dm
CPc.d.dm
RSP
VIS
PTLp
MMme
ENTm

Supplementary Fig. 6. IAD connections with CP

a-b. An anterograde tracer in the AM labels terminals in the dorsomedial CP both at the rostral (CPi.dm, a) and caudal (CPc.dm, b) levels. Retrograde tracers placed in the CPi.dm and CPc.dm show labeled projection neurons in the IAD disclosing the IAD→CPi.dm/CPc.dm connections. **c.** Repeated retrograde FG injections into the CPi.dm and intermediate domains (CPi.im) revealed retrograde labeling in the IAD, but not in AM, confirming IAD→CPi.dm projections (see Supplementary Table 2 for number of repeated injections made in each ROI). **d.** Cre-dependent anterograde tracing of AMd/IAD neurons (Cre-dependent AAV-GFP) compared to Cre-dependent tracing of AM neurons that do not involve the IAD (Cre-dependent AAV-RFP) show the former labeling the CPi.dm and CPc.dm (green), whereas the latter injection that does not trace IAD neurons does not (red). Cre was delivered to the AM through a large AAVretro-Cre injection that affected the ILA, PL, and ACA. **e.** Quantification of the normalized projection density in the CP following Cre-dependent injections into the IAD/AMd, AMd.medial (AMd.m), and the AMv validates the IAD→CP connection. **f.** The detailed CPi (top) and CPc (bottom) dorsomedial domains are visualized atop the projections from the IAD. All ROIs that project to the CPi and CPc dorsomedial domains are presented to demonstrate the region's role in integrating visual and spatial information. **g.** Anterograde AAV tracer injections into the SC revealed axonal labeling in the IAD suggesting an SC→IAD connection. **h.** Schematic of IAD in a network of ROIs involved in visual and spatial processing. Note that the CPi.dm (CPi.dm.d, CPi.dm.dm) and CPc.dm (CPc.d.dm) domains that the IAD projects to are the domains that integrate converging visual/spatial information from the ACAv, MOs-fef, ENTm, RSP, VIS, and PTLp. The inputs of each domain are listed. See Supplementary Table 2 for structure abbreviations.

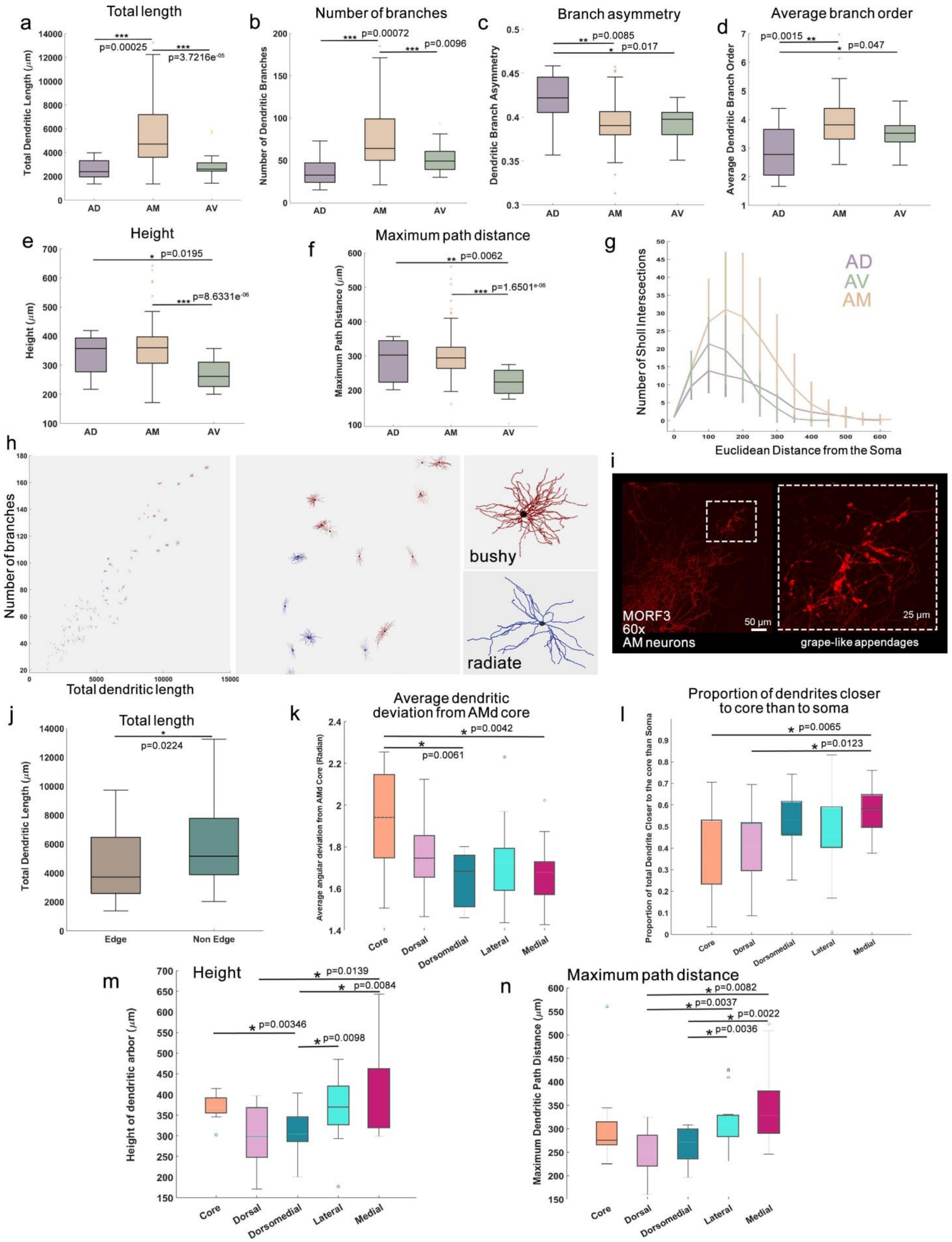
a
Total length
Total Dendritic Length (μm)
p=0.00025
p=3.7216e-05
AD
AM
AV
b
Number of branches
Number of Dendritic Branches
p=0.00072
p=0.0096
c
Branch asymmetry
Dendritic Branch Asymmetry
p=0.0085
p=0.017
d
Average branch order
Average Dendritic Branch Order
p=0.0015
p=0.047
e
Height
Height (μm)
p=0.0195
p=8.6331e-06
f
Maximum path distance
Maximum Path Distance (μm)
p=0.0062
p=1.6501e-06
g
Number of Sholl Intersections
Euclidean Distance from the Soma
AD
AV
AM
h
Number of branches
Total dendritic length
bushy
radiate
i
MORF3
60x
AM neurons
50 μm
25 μm
grape-like appendages
j
Total length
Total Dendritic Length (μm)
p=0.0224
Edge
Non Edge
k
Average dendritic deviation from AMd core
Average angular deviation from AMd Core (Radian)
p=0.0042
p=0.0061
Core
Dorsal
Dorsomedial
Lateral
Medial
l
Proportion of dendrites closer to core than to soma
Proportion of total Dendrite Closer to the core than Soma
p=0.0065
p=0.0123
m
Height
Height of dendritic arbor (μm)
p=0.0139
p=0.0084
p=0.00346
p=0.0098
n
Maximum path distance
Maximum Dendritic Path Distance (μm)
p=0.0082
p=0.0037
p=0.0022
p=0.0036

Supplementary Fig. 7, related to Fig. 10. Dendritic morphological comparison of ATN neurons
Boxplot comparisons between AD (n=10), AM (n=82), and AV (n=17) neurons in **a,** total dendritic length, **b,** total number of branches, **c,** branch asymmetry, **d,** average branch order, **e,** height, and **f,** maximum dendritic path distance. Two-sided Wilcoxon signed rank test was used for groupwise comparisons, and False Discovery Rate (FDR) corrections were carried out for multiple comparisons. **g.** Sholl intersection profile (number of dendritic intersections per concentric sphere drawn at 50 μm intervals) shows a larger number of intersections for AM neurons compared to AV and AD neurons. AV neurons have a higher peak compared to AD neurons, whereas AD neurons extend further and have more intersections away from the cell body than AV neurons. **h.** All ATN neurons (n=109) classified into two morphological types based on total dendritic wiring length and number of branches are plotted in the left-most. Middle panel magnifies the boundary between the morphological clusters where Type 1 (blue) neurons are smaller, less complex and Type 2 (red) are larger, more complex. Right-most panel shows representative Type 1 and Type 2 neurons that resemble previously characterized radiate (blue) and bushy (red) neurons. **i.** AM neurons show grape-like appendages previously identified in thalamic neurons. See Supplementary Table 2 for structure abbreviations. **j.** Boxplot comparison shows greater dendritic length between AMd non-edge neurons (n=45) compared to edge neurons (n=27). Two-sided t-test used for groupwise comparisons with FDR correction [t(70)=-2.334, p=0.022441]. **k-n**. Boxplot comparisons between AMd.dorsal (n=12), AMd.dorsomedial (n=11), AMd.medial (n=17), AMd.lateral (n=20), and AMd.core (n=12) neurons in **k,** average dendritic deviation from AMd center, **l,** proportion of dendritic arbors closer to AMd center than soma, **m,** height, and **n,** maximum dendritic path length. Two-sided Wilcoxon signed rank test was used for groupwise comparisons, and FDR corrections were carried out for all comparisons. For all comparisons, traced AM, AD, and AV neurons were collected across independent cases and aggregated for analysis. The line inside each boxplot is the sample median. Top and bottom edges are upper and lower quartiles, respectively. The distance between the top and bottom edges is the interquartile range (IQR). Upper quartile corresponds to the 0.75 quantile and lower quartile corresponds to the 0.25 quantile. Outliers ('o') are values that are more than 1.5 IQR away from the top or bottom of the box. Top whisker connects upper quartile to the nonoutlier maximum, and the other connects lower quartile to the nonoutlier minimum. *= $p \leq 0.05$, **= $p \leq 0.01$), and ***= $p \leq 0.001$.

Supplementary Table 1

Region of Interest (ROI)	Number of injections
Anterior cingulate cortical area, dorsal part (ACAd)	6
Anterior cingulate cortical area, ventral part (ACAv)	8
Anterodorsal nucleus of the thalamus (AD)	2
Anteromedial nucleus of the thalamus (AMd)	24
Anteromedial nucleus of the thalamus, ventral part (AMv)	2
Anteroventral nucleus of the thalamus (AV)	5
Basolateral amygdalar nucleus, anterior part (BLAa)	2
Caudoputamen, caudal part, dorsomedial region (CPc.dm)	6
Caudoputamen, intermediate part, dorsomedial region (CPi.dm)	5
Dorsal tegmental nucleus (DTN)	3
Entorhinal cortical area, lateral part (ENTl)	12
Entorhinal cortical area, medial part (ENTm)	7
Infralimbic cortical area (ILA)	10
Laterodorsal tegmental nucleus (LDT)	4
Lateral mammillary nucleus (LM)	2
Medial mammillary nucleus, lateral part (MMl)	2
Medial mammillary nucleus, medial part (MMm)	4
Medial mammillary nucleus, median part (MMme)	1
Secondary motor cortical area (MOs)	14
Secondary motor cortical area, frontal eye field region (MOs-fef)	3
Orbital cortical area, lateral part (ORBl)	1
Orbital cortical area, medial part (ORBm)	3
Orbital cortical area, ventrolateral part (ORBvl)	2
Parasubiculum (PAR)	3
Prelimbic cortical area (PL)	11
Postsubiculum (POST)	3
Presubiculum (PRE)	7
Posterior parietal association area, caudal lateral part (PTLp c, l)	2
Posterior parietal association area, caudal medial part (PTLp c, m)	5
Retrosplenial cortical area, agranular part (RSPagl)	2
Retrosplenial cortical area, dorsal part (RSPd)	3
Retrosplenial cortical area, ventral part (RSPv)	9
Superior colliculus (SC)	5
Subiculum, dorsal part (SUBd)	8
Subiculum, ventral part (SUBv)	7
Ventral tegmental nucleus (VTN)	3

Supplementary Table 1. Injections. The number of injections that were made in each region of interest.

Supplementary Table 2

Acronym	Full structure name
AAV	Adeno-associated virus
AAV-GFP	Adeno-associated virus, green fluorescent protein
AAV-RFP	Adeno-associated virus, red fluorescent protein
ac	Anterior commissure
ACAd	Anterior cingulate cortical area, dorsal part
rACAd	Anterior cingulate cortical area, dorsal part, rostral region
cACAd	Anterior cingulate cortical area, dorsal part, caudal region
ACAv	Anterior cingulate cortical area, ventral part
rACAv	Anterior cingulate cortical area, ventral part, rostral region
cACAv	Anterior cingulate cortical area, ventral part, caudal region
ACB	Nucleus accumbens
AD	Anterodorsal nucleus of the thalamus
AD.l	Anterodorsal nucleus of the thalamus, lateral domain
AD.m	Anterodorsal nucleus of the thalamus, medial domain
AM	Anteromedial nucleus of the thalamus
AMd	Anteromedial nucleus of the thalamus, dorsal part
AMd.c	Anteromedial nucleus of the thalamus, dorsal part, core domain
AMd.d	Anteromedial nucleus of the thalamus, dorsal part, dorsal domain
AMd.dl	Anteromedial nucleus of the thalamus, dorsal part, dorsolateral domain
AMd.dm	Anteromedial nucleus of the thalamus, dorsal part, dorsomedial domain
AMd.l	Anteromedial nucleus of the thalamus, dorsal part, lateral domain
AMd.m	Anteromedial nucleus of the thalamus, dorsal part, medial domain
AMv	Anteromedial nucleus of the thalamus, ventral part
AV	Anteroventral nucleus of the thalamus
AV.d	Anteroventral nucleus of the thalamus, dorsal domain
AV.l	Anteroventral nucleus of the thalamus, lateral domain
AV.m	Anteroventral nucleus of the thalamus, medial domain
AV.mt	Anteroventral nucleus of the thalamus, medial tip domain
AQ	Cerebral aqueduct
BLA	Basolateral amygdalar complex
BLAa	Basolateral amygdalar nucleus, anterior part
BLA.ac	Basolateral amygdalar nucleus, anterior part, caudal domain
BLAp	Basolateral amygdalar nucleus, posterior part
BLAv	Basolateral amygdalar nucleus, ventral part

BMAp	Basomedial amygdalar nucleus, posterior part
BST	Bed nuclei of the stria terminalis
CEA	Central amygdalar nucleus
CM	Central medial nucleus of the thalamus
CP	Caudoputamen
CPc	Caudoputamen, caudal part
CPc.dm	Caudoputamen, caudal part, dorsomedial region
CPc.dm.d	Caudoputamen, caudal part, dorsomedial region, dorsal domain
CPi	Caudoputamen, intermediate part
CPi.dm	Caudoputamen, intermediate part, dorsomedial region
CPi.dm.d	Caudoputamen, intermediate part, dorsomedial region, dorsal domain
CPi.dm.dm	Caudoputamen, intermediate part, dorsomedial region, dorsomedial domain
CPi.vm	Caudoputamen, intermediate part, ventromedial region
CTB	Cholera toxin subunit B
DR	Dorsal raphe nucleus
DTN	Dorsal tegmental nucleus
ECT	Ectorhinal cortical area
rECT	Ectorhinal cortical area, rostral region
cECT	Ectorhinal cortical area, caudal region
ENT	Entorhinal cortical area
ENTl	Entorhinal cortical area, lateral part
cENTl	Entorhinal cortical area, lateral part, caudal region
rENTl	Entorhinal cortical area, lateral part, rostral region
ENTm	Entorhinal cortical area, medial part
FG	Fluorogold
IAD	Interanterodorsal nucleus of the thalamus
IAM	Interanteromedial nucleus of the thalamus
ILA	Infralimbic cortical area
IMD	Intermediodorsal nucleus of the thalamus
int	Internal capsule
LA	Lateral amygdalar area
LD	Laterodorsal nucleus of the thalamus
LDT	Laterodorsal tegmental nucleus
LM	Lateral mammillary nucleus
LS	Lateral septum
MD	Mediodorsal nucleus of the thalamus

MDm	Mediodorsal nucleus of the thalamus, medial part
MEA	Medial amygdalar nucleus
MM	Medial mammillary nucleus
MMl	Medial mammillary nucleus, lateral part
MMm	Medial mammillary nucleus, medial part
MMmed	Medial mammillary nucleus, median part
MOs	Secondary motor cortical area
MOs fef	Secondary motor cortical area, frontal eye field region
ORB	Orbital cortical area
ORBl	Orbital cortical area, lateral part
ORBm	Orbital cortical area, medial part
ORBvl	Orbital cortical area, ventrolateral part
OT	Olfactory tubercle
PAR	Parasubiculum
PERI	Perirhinal cortical area
rPERI	Perirhinal cortical area, rostral region
cPERI	Perirhinal cortical area, caudal region
PF	Parafascicular nucleus of the thalamus
PH	Posterior hypothalamic nucleus
PHAL	*Phaseolus vulgaris* leucoagglutinin
PL	Prelimbic cortical area
POST	Postsubiculum
PRE	Presubiculum
PT	Parataenial nucleus of the thalamus
PTLp	Posterior parietal association area
PTLp cl	Posterior parietal association area, caudal lateral part
PTLp cm	Posterior parietal association area, caudal medial part
PVT	Paraventricular nucleus of the thalamus
rPVT	Paraventricular nucleus of the thalamus, rostral region
cPVT	Paraventricular nucleus of the thalamus, caudal region
RE	Reuniens nucleus of the thalamus
RH	Rhomboid nucleus of the thalamus
RSP	Retrosplenial cortical area
RSPagl	Retrosplenial cortical area, agranular part
RSPd	Retrosplenial cortical area, dorsal part
RSPv	Retrosplenial cortical area, ventral part
RT	Reticular nucleus of the thalamus

SC	Superior colliculus
sm	Stria medularis
SNr	Substantia nigra, pars reticulata
SUB	Subiculum
SUBd	Subiculum, dorsal part
SUBv	Subiculum, ventral part
TEa	Temporal association cortical area
rTEa	Temporal association cortical area, rostral region
cTEa	Temporal association cortical area, caudal region
VIS	Visual cortical area
VISal	Anterolateral visual cortical area
VISam	Anteromedial visual cortical area
VISl	Lateral visual cortical area
VISp	Primary visual cortical area
VISpm	Posteromedial visual cortical area
VTA	Ventral tegmental area
VTN	Ventral tegmental nucleus
ZI	Zona incerta

Supplementary Table 2. Brain structure abbreviations. The table provides all the abbreviations that were used for both brain structures and tracers. Lower case, italicized acronyms denote fiber tracts.
