# Supplementary material for: Distinct subnetworks of the mouse anterior thalamic nuclei
Source: Nat Commun. 2025 Jul 1;16:6018. doi: 10.1038/s41467-025-60774-6 (PMC12216302; doi:10.1038/s41467-025-60774-6)
Supplement: Supplementary file 2 — Reporting Summary [file 41467_2025_60774_MOESM2_ESM.pdf]

nature portfolio

Corresponding author(s): Houri Hintiryan, Hong-Wei Dong

Last updated by author(s): 5/8/2024

Reporting Summary

Nature Portfolio wishes to improve the reproducibility of the work that we publish. This form provides structure for consistency and transparency in reporting. For further information on Nature Portfolio policies, see our Editorial Policies and the Editorial Policy Checklist.

Statistics

For all statistical analyses, confirm that the following items are present in the figure legend, table legend, main text, or Methods section.

n/a	Confirmed	
[ ]	[x]	The exact sample size (*n*) for each experimental group/condition, given as a discrete number and unit of measurement
[ ]	[x]	A statement on whether measurements were taken from distinct samples or whether the same sample was measured repeatedly
[ ]	[x]	The statistical test(s) used AND whether they are one- or two-sided<br>*Only common tests should be described solely by name; describe more complex techniques in the Methods section.*
[x]	[ ]	A description of all covariates tested
[ ]	[x]	A description of any assumptions or corrections, such as tests of normality and adjustment for multiple comparisons
[ ]	[x]	A full description of the statistical parameters including central tendency (e.g. means) or other basic estimates (e.g. regression coefficient) AND variation (e.g. standard deviation) or associated estimates of uncertainty (e.g. confidence intervals)
[ ]	[x]	For null hypothesis testing, the test statistic (e.g. *F*, *t*, *r*) with confidence intervals, effect sizes, degrees of freedom and *P* value noted<br>*Give P values as exact values whenever suitable.*
[x]	[ ]	For Bayesian analysis, information on the choice of priors and Markov chain Monte Carlo settings
[x]	[ ]	For hierarchical and complex designs, identification of the appropriate level for tests and full reporting of outcomes
[x]	[ ]	Estimates of effect sizes (e.g. Cohen's *d*, Pearson's *r*), indicating how they were calculated

Our web collection on statistics for biologists contains articles on many of the points above.

Software and code

Policy information about availability of computer code

Data collection	No commercial or custom code was used for data collection.
Data analysis	The code used to analyze the data for this project can be found here: https://github.com/ucla-brain/atn_community.

For manuscripts utilizing custom algorithms or software that are central to the research but not yet described in published literature, software must be made available to editors and reviewers. We strongly encourage code deposition in a community repository (e.g. GitHub). See the Nature Portfolio guidelines for submitting code & software for further information.

Data

Policy information about availability of data

All manuscripts must include a data availability statement. This statement should provide the following information, where applicable:

- Accession codes, unique identifiers, or web links for publicly available datasets
- A description of any restrictions on data availability
- For clinical datasets or third party data, please ensure that the statement adheres to our policy

Source data are provided in a Source Data file.

Research involving human participants, their data, or biological material

Policy information about studies with human participants or human data. See also policy information about sex, gender (identity/presentation), and sexual orientation and race, ethnicity and racism.

Reporting on sex and gender	N/A
Reporting on race, ethnicity, or other socially relevant groupings	N/A
Population characteristics	N/A
Recruitment	N/A
Ethics oversight	N/A

Note that full information on the approval of the study protocol must also be provided in the manuscript.

Field-specific reporting

Please select the one below that is the best fit for your research. If you are not sure, read the appropriate sections before making your selection.

☒ Life sciences ☐ Behavioural & social sciences ☐ Ecological, evolutionary & environmental sciences

For a reference copy of the document with all sections, see nature.com/documents/nr-reporting-summary-flat.pdf

Life sciences study design

All studies must disclose on these points even when the disclosure is negative.

Sample size	150 8-week-old C57Bl/6J (Jackson Laboratories) male mice were used (see Methods).<br>17 male MORF3 mice were used from our established breeding colony at UCLA (see Methods).<br>Supplementary Table 1 shows the number of repeated injections made in each ROI presented in the paper.<br>The number of neurons analyzed for all comparisons is also included both in the text and the corresponding figure legends.
Data exclusions	In a typical neuroanatomy experiment, cases with missed injection sites are used as controls and are not excluded.<br>For neuron reconstructions, neurons that had non-optimal tracing or imaging were not reconstructed.
Replication	All of our connectivity data are rigorously validated to ensure reliability to the extent possible. This includes making repeated injections in the same ROIs (presented in Supplementary Table 1) and using different injection strategies that are discussed throughout the paper. We have also included a Data Reproducibility section in the Methods to address replication of the data.
Randomization	Randomization was not performed for the neural tracing experiments since animals were not assigned to different groups and the data were not statistically compared. Randomization is necessary for reducing bias and controlling variability. Instead, the data were validated in different ways (see Data Reproducibility section).<br>Randomization was also not necessary for the morphological comparisons since due to the characteristics of MORF3, we achieve random labeling of neurons and then reconstruct the traceable neurons.
Blinding	For tracing experiments, blinding was not necessary because animals were not assigned to different groups. For neuron reconstructions, technicians were not blinded while digitally reconstructing the neurons since we achieve random labeling of neurons with MORF and then reconstruct the ones with good image in our regions of interest.

Reporting for specific materials, systems and methods

We require information from authors about some types of materials, experimental systems and methods used in many studies. Here, indicate whether each material, system or method listed is relevant to your study. If you are not sure if a list item applies to your research, read the appropriate section before selecting a response.

Materials & experimental systems

n/a	Involved in the study	
[ ]	[x]	Antibodies
[x]	[ ]	Eukaryotic cell lines
[x]	[ ]	Palaeontology and archaeology
[ ]	[x]	Animals and other organisms
[x]	[ ]	Clinical data
[x]	[ ]	Dual use research of concern
[x]	[ ]	Plants

Methods

n/a	Involved in the study	
[x]	[ ]	ChIP-seq
[x]	[ ]	Flow cytometry
[x]	[ ]	MRI-based neuroimaging

Antibodies

Antibodies used

Primary antibodies used
anti-PHAL (1:1000 rabbit anti-Phal antibody, Vector Laboratories, #AS-2300)
anti-Cre (1:4000 mouse anti-Cre recombinase antibody, EMD Millipore, #MAB3120)
anti-V5 [1:1000 anti-V5, Fortis, #A190-119A (goat) or #A190-120A (rabbit)]

Secondary antibodies used
For PHAL (1:500 concentration of anti-rabbit IgG conjugated with Alexa Fluor® 488 or 647; Invitrogen, 488: #A-21206; 647: #A-31573)
For Cre (1:500 concentration of anti-mouse IgG conjugated with Alexa Fluor® 488 or 647; Life Technology, 488: #A-21202; 647: #A-31571)
For V5 (1:1000 in house conjugated Fab-Setau-647, details described in the Methods section)

Cytoarchitectural background staining
NeuroTrace 435/455 (1:500; Invitrogen, #N21479)
DAPI (1:500, ThermoFisher Scientific, #D1306)
Syto 13 (1:500, ThermoFisher Scientific, #S7575)
Propidium iodide (1:500, ThermoFisher Scientific, #P1304MP)

Validation

We have extensively used each of these antibodies and have demonstrated through our publications that they all work well (Zingg et al., 2014; Hintiryan et al., 2016; Bienkowski et al., 2018; Foster et al., 2021; Hintiryan et al., 2021; Benavidez et al., 2021).

Animals and other research organisms

Policy information about studies involving animals; ARRIVE guidelines recommended for reporting animal research, and Sex and Gender in Research

Laboratory animals

150 8-week-old C57Bl/6J (Jackson Laboratories) male mice were used (stated in the Methods section).
17 male MORF3 mice were used from our established breeding colony at UCLA (stated in the Methods section).

Wild animals

N/A

Reporting on sex

Only male mice were utilized in this study. To our knowledge, sexually dimorphic connections are challenging to identify at the mesoscale resolution, predominantly employed here, particularly in the thalamus compared to more sexually dimorphic brain regions like the hypothalamus. Although we previously examined sex differences in mesoscale connections in certain brain areas, such as the basolateral amygdala, we did not observe any notable differences. Importantly, we aim to consolidate all our connectome research to establish the most comprehensive and reliable brain-wide mammalian connectome. Given that most of our connectivity experiments are conducted using male mice, maintaining consistency in our approach was essential.

Field-collected samples

N/A

Ethics oversight

All procedures were conducted in compliance with regulatory standards outlined in the National Institutes of Health Guide for the Care and Use of Laboratory Animals and institutional guidelines established by the Institutional Animal Care and Use Committee at the University of Southern California (USC) and at the University of California, Los Angeles (UCLA).

Note that full information on the approval of the study protocol must also be provided in the manuscript.

Plants

Seed stocks	N/A
Novel plant genotypes	N/A
Authentication	N/A
